# Purification and quantitative proteomic analysis of cell bodies and protrusions

**DOI:** 10.1016/j.xpro.2021.100462

**Published:** 2021-04-10

**Authors:** Maria Dermit, Faraz K. Mardakheh

**Affiliations:** 1 Centre for Cancer Cell and Molecular Biology, Barts Cancer Institute, Queen Mary University of London, Charterhouse Square, London EC1M 6BQ, UK

**Keywords:** Bioinformatics, Cell biology, Cell separation/fractionation, Proteomics, Mass spectrometry

## Abstract

Actin-rich protrusions are membrane extensions generated by actin polymerization that drive mesenchymal-like cell migration. Characterization of protrusions proteome is crucial for understanding their function. We present a complete step-by-step protocol based on microporous filter-based fractionation of protrusive cellular domains coupled with sample preparation for quantitative proteomics, mass spectrometric data acquisition, and data analysis. This protocol enables purification, quantification, and analysis of the distribution of proteins present in protrusions and cell bodies.

For complete details on the use and execution of this protocol, please refer to Dermit et al. (2020).

## Highlights

• A protocol for quantitative analysis of protrusion proteomes
• Cell bodies and protrusions are isolated by transwell fractionation
• Fractions are analyzed by tandem mass tagging-based shotgun proteomics
• A step-by-step pipeline is provided for data analysis

## Graphical abstract

**Figure:**
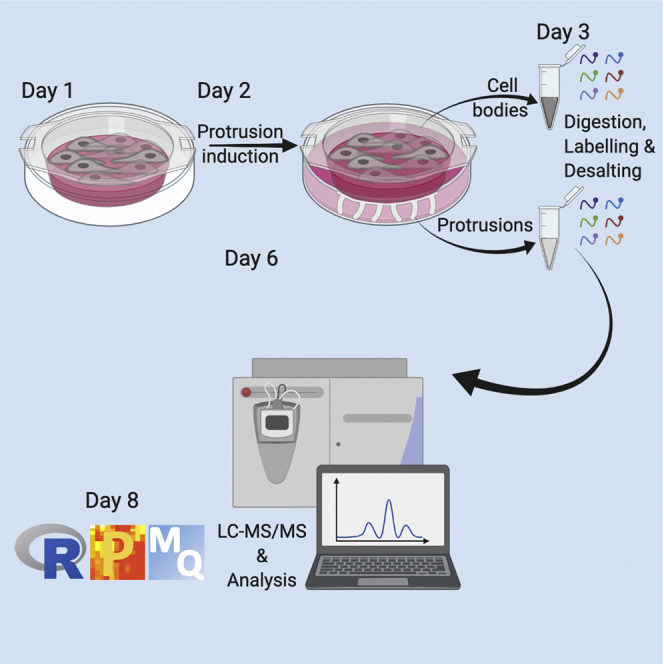

## Before you begin

**Figure 1:**
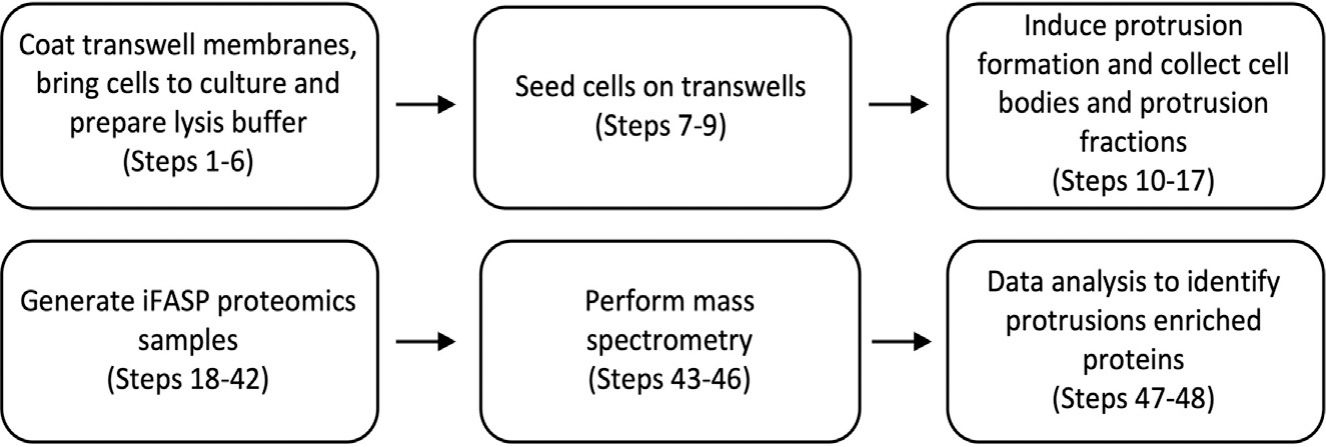
Workflow for performing quantitative proteomics analysis of protrusions and cell bodies Transwell membranes should be collagen-I coated and number of cells to reach 100% confluency on Transwells should be optimized. After cells are allowed to form protrusions for a given time (30 min, 60 min, 120 min, 240 min and 480 min) cell bodies and protrusion are collected, proteomics samples are generated for mass spectrometry analysis and data analysis is performed to identify enriched proteins

***Note:*** Please see Figure 1 for an overview of the complete workflow.

### Coat Transwells

**Timing: 2.5 h**

***Note:*** Work in sterile conditions (typically a biosafety hood).

1. Prepare 5 μg/mL collagen-I solution in sterile double-distilled tissue culture compatible water (ddH_2_O). Keep on ice.
2. Add 8 mL of collagen-I solution to the top of 75 mm Transwell filters with 3-μm pore size and 12 mL of collagen-I solution to bottom. Leave the solution for 2 h at 20°C–23°C to coat both sides of the filter.
3. Remove the solution completely and allow the filter membranes to dry (30–60 mins).
4. Make sure the membrane is fully dried. Fully dried membranes turn opaque. Close the Transwell and seal with parafilm. Store Transwell filter at 4°C until use.

***Note:*** Coated Transwell filters can be stored for few weeks at 4°C.

***Note:*** The minimum amount of protein required for this protocol is 25 μg (see Bicinchoninic acid [BCA] protein assay on step 12). The amount of protrusions collected may vary across cell lines. If the amount of protrusion is not enough from one 75 mm filter plate, material from individual plates may be pooled.

### Bring cells into culture

**Timing: ∼ 3 days**

5. If working with cell lines, a few days before the start of the experiment (day -4 or-3), bring the cells of interest to culture, and propagate cells on appropriate culture media for acclimation. The total number of cells needed for one 75 mm filter experiment is 10,000,000.

***Note:*** We mostly used MDA-MB231 cells for our studies. These cells are grown in DMEM supplemented with 10% Fetal Bovine Serum (FBS), 1% Penicillin/Streptomycin.

***Note:*** If possible, perform mycoplasma test and short tandem repeat (STR) profiling of the cells beforehand to guarantee culture is mycoplasma free and cells are validated. The cells of our study were authenticated by STR profiling (Public Health England) and were routinely checked to be mycoplasma-free biweekly.

***Note:*** All cell incubations are performed at 37°C in a CO_2_ incubator. This requirement may be different for other cell lines.

**Figure 2:**
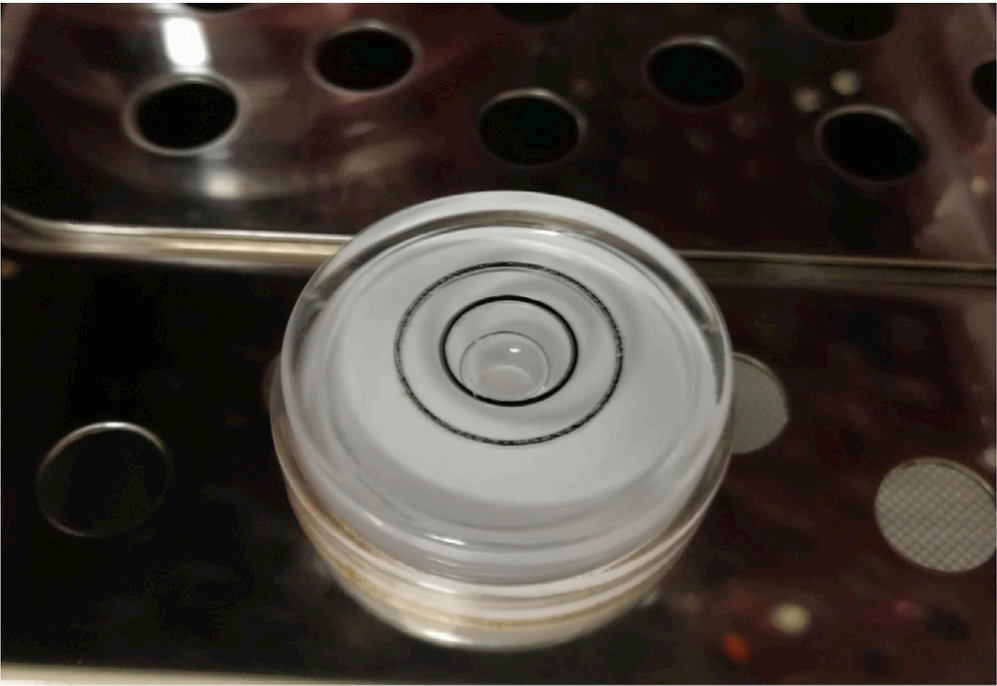
Measurement of cell incubator balance using a spirit level Spirit level indicates how parallel the incubator shelf is relative to the earth. The gas bubble centered inside the inner circle of the round bullseye precision spirit level indicates the incubator shelf is level.

**CRITICAL:** Ensure that the shelving in your incubator is perfectly straight. Unlevel shelves will cause media leakage from top of microporous filters to the bottom chamber at incorrect timing. You can use a spirit level to rule out any slight inclination in your incubator as shown in Figure 2.

### Prepare buffers

6. Prepare 10 mM Tris-HCl lysis buffer, pH 7.5 containing 2% SDS (Sodium dodecyl sulfate) to collect protrusions and cell-bodies (See materials and equipment) (Mardakheh et al., 2015).

## Key resources table

**Table:** 

REAGENT/RESOURCE	SOURCE	IDENTIFIER
**Chemicals, peptides, and recombinant proteins**		

Collagen I	Advanced BioMatrix	Cat#5005
DMEM (to grow MDA-MB231 cells)	Sigma	Cat#6429
FBS	Fisher Scientific	Cat#11306060
Penicillin/streptavidin	Fisher Scientific	Cat#15140122
Cover slips	Thermo Fisher Scientific	Cat#222215
Bond-Breaker™ TCEP Solution	Thermo Fisher Scientific	Cat#77720
Iodoacetamide	VWR Chemicals	Cat#786-228
Urea	Sigma	Cat#U1250
SDS	Severn Biotech Ltd	Cat#20-4000-10
Tris-HCl pH 7.5	Severn Biotech Ltd	Cat#20-7901-10
Tris-HCl pH 8.5	Severn Biotech Ltd	Cat#20-7900-01
Acetonitrile LC-MS Grade	Thermo Fisher Scientific	Cat# A/0638/17
Sequencing grade trypsin	Sigma	Cat#11418475001
Quenching reagent (50% hydroxylamine)	Thermo Fisher Scientific	Cat#90115

**Critical commercial assays**		

TMT10plex™ Isobaric Label Reagent Set	Thermo Fisher Scientific	Cat#90110
Pierce High pH Reversed-Phase Peptide Fractionation Kit	Life Technologies	Cat#84868
Pierce™ BCA Protein Assay Kit	Thermo Fisher Scientific	Cat#23225
Formic acid Optima™ LC/MS Grade	Thermo Fisher Scientific	Cat#A117-50
Trifluoroacetic acid ULC- MS Optigrade®	Promochem	Cat#SO-9668-B001
MycoAlert PLUS Mycoplasma Detection Kit	Lonza	Cat#LT07-703
Methanol	Fisher Scientific	Cat#10214490
PBS	Sigma	Cat#P4417

**Deposited data**		

TMT quantitative proteomics analysis of protrusions and cells bodies of MDA-MB231 cells collected after 30 min, 1 h, 2 h, 4 h, or 8 h after induction of protrusions on 3-μm pore Transwells	N/A	PXD015808 accessible via PRIDE partner repository (http://www.ebi.ac.uk/ pride/archive/)

**Experimental models: cell lines**		

MDA-MB231	ATCC	ATCC number 92020424

**Software and algorithms**		

MaxQuant 1.6.3.3	N/A	https://www.biochem.mpg.de/5111795/maxquant
Perseus 1.5.5.3	N/A	https://www.biochem.mpg.de/5111810/perseus
R 4.1	N/A	https://www.R-project.org/

**Other**		

75 mm Transwell® with 3.0 μm pore polycarbonate membrane insert	Corning	Cat#3420
Vivacon® 500 centrifugal concentrator spin columns with 30 kDa molecular weight cutoff filters	Sartorius	Cat#VN01H21
Eppendorf® LoBind microcentrifuge tubes 1.5 mL	Sigma	Cat#Z666505
Eppendorf® LoBind microcentrifuge tubes 2 mL	Sigma	Cat#Z666513
Clear Autosampler Vial Kits	Thermo Fisher Scientific	Cat#C5000-192W
Micro centrifuge for 1.5–2.0 mL tubes	Multiple suppliers available	N/A
Cell incubator	Multiple suppliers available	N/A
Aspirator	Multiple suppliers available	N/A
Vortex	Multiple suppliers available	N/A
Bioruptor Pico sonication device	Diagenode	Cat# B01060003
FLUOstar Omega microplate reader	BMG Labtech	Cat#415-102-AFL
Thermomixer comfort for 1.5 mL tubes	Eppendorf	Cat#5355
SpeedVac	Thermo Fisher Scientific	Cat#SPD1010
Q-Exactive™ Plus Orbitrap Mass Spectrometer	Thermo Fisher Scientific	Cat#IQLAAEGAAPFALGMBCA
Personal computer with minimum requirements to run MaxQuant (http://www.coxdocs.org/doku.php?id=maxquant:common:download_and_installation#:∼:text=exe%20mqpar.xml-,Hardware%20requirements,on%20the%20number%20of%20cores.)	Multiple suppliers available	N/A

## Materials and equipment

***Alternatives:*** This protocol describes purification of MDA-MB231 cell bodies and protrusions using 75 mm filters. We use polycarbonate Transwells, which are compatible with organic fixatives such as methanol. However, smaller Transwells (e.g., 24 mm inserts) can be used and material pooled together. We have not evaluated the influence of other synthetic membrane materials such as polyester or mammalian/non-mammalian collagen coating materials on protrusion proteomes.

***Alternatives:*** This protocol describes protrusion purification for MDA-MB231 cells. Alternative cell lines may be used, and cell number may be optimized to warrant 100% confluency. Confluency can be checked with microscopy, staining cells with either DAPI or phalloidin. For cell number optimization smaller Transwells (e.g., 24 mm) could be used.

***Alternatives:*** This protocol uses an automated cell counter (Countess™ II), but other cell counter methods may be used.

***Alternatives:*** This protocol estimates protein amounts by commercial Pierce BCA Protein Assay Kit, prior to sample preparation for liquid chromatography-tandem mass spectrometry (LC-MS/MS). Other protein estimation methods that are compatible with SDS can be used.

***Alternatives:*** This protocol uses Tandem Mass Tag™ (TMT™) 10-plex-based protein quantitation because isobaric approaches increase sample throughput and reduce missing quantitative values (Werner et al., 2012). Other quantitative approaches such as SILAC have also been used successfully, for instance (Mardakheh et al., 2015).

***Alternatives:*** This protocol uses a nanoflow ultimate 3000 RSL nano HPLC platform (Thermo Fisher). An LC system that can deliver nanoflow rates and can operate up to a pressure of 1,000 bar could be used for peptide separation.

***Alternatives:*** This protocol uses a Q-Exactive™ Plus mass spectrometer. Other LC-MS/MS systems can be used as long as they have sufficient resolution in MS/MS mode to resolve the low-mass N and C series TMT10plex reporter ions. To accurately measure reporter ions in the TMT10plex reagent, the instrument minimum MS/MS resolution must be set at 35,000.

***Alternatives:*** This protocol describes proteomics data analysis with MaxQuant and Perseus. These free tools are one of the most frequently used platforms for mass-spectrometry (MS)- based proteomics data analysis. MaxQuant platform includes a database search engine (Andromeda) to perform peptide identification and algorithms and tools designed for label-free, MS1-level labelling, and isobaric labelling-based quantitation. MaxQuant and Perseus are freely distributed, although subject to license terms (See https://www.maxquant.org/download_asset/maxquant/latest and https://www.maxquant.org/download_asset/perseus/latest). Analysis of MS data could be done with other software packages that support the identification and quantification of TMT10plex reporter ions. We developed an open source R package extension called *protrusionproteome* that includes the data used in this manuscript, as well as the functions that allow the analytical workflow described in the method paper. *protrusionproteome* is available for download at https://github.com/demar01/protrusionproteome.

**Table:** Lysis buffer

Reagent	Final concentration	Stock concentration	Add to 10 mL
SDS	2% (v/v)	10%	2 mL
Tris-HCl pH 7.5	100 mM	1 M	1 mL
MiliQ water	N/A	N/A	7 mL

This solution can be stored at 20°C–22°C for several months.

**Table:** Urea (UA) buffer

Reagent	Final concentration	Stock concentration	Add to 40 mL
Urea	8 M	Pellets (60.06 gr/mol)	19.2 gr
Tris HCl pH 8.5	100 mM	1.5 M	2666 μL
MiliQ water	N/A	N/A	Up to 40 mL

**CRITICAL:** Prepare fresh on the day of use (Day 3). The amount of UA buffer per sample is ∼1–5 mL. Here we prepare 40 mL for 10 samples.

**Table:** Dissolution buffer

Reagent	Final concentration	Stock concentration	Add to 5 mL
TEAB	100 mM	1 M	0.5 mL
MiliQ water	N/A	N/A	4.5 mL

Prepare on Day 3

## Step-by-step method details

### Seeding cells on Transwells - day 1

**Timing: 16 h**

Cells are seeded on top of Transwells and allowed to adhere.

1. If working with adherent cells (like MDA-MB231 cells), initially trypsinize cells. See Table 1 for steps followed to detach cells from the tissue culture plates.

**Table 1:** .Steps to trypsinize cells

Step	Action
1	Remove culture media from flask
2	Wash flask twice with PBS
3	Add 0.05% EDTA-trypsin and incubate for 5 min at 37°C to allow cell detaching
4	Inactivate EDTA-trypsin by adding approximately three volumes of media
5	Centrifugation mix at 300–1000 *g* for 5 min at 20°C–23°C
6	Resuspend cell pellets in fresh media
2. Once the cells are trypsinized and resuspended in fresh media, count cells with an automated cell counter.
3. Carefully seed 10 million cells resuspended in 3.5 mL of culture media on top of collagen-I coated filter, and allow them to adhere to the filter for about 16 h in the incubator (See Methods video S1 and Figure 3). Do not add media to the bottom chamber.

**CRITICAL:** 3.5 mL is enough to cover a 75 mm microporous filter. Make sure the media is distributed homogeneously across the filter to warrant a homogenous cell monolayer. Adding more media to the top of filter may cause media leakage from chamber top to bottom troubleshooting 1.

***Note:*** If required, perform an additional step to concentrate the cells by centrifugation.

***Note:*** We allow cell attachment to filter for about 16 h. Depending on the cell type, shorter attachment times could be used.

**Figure 3:**
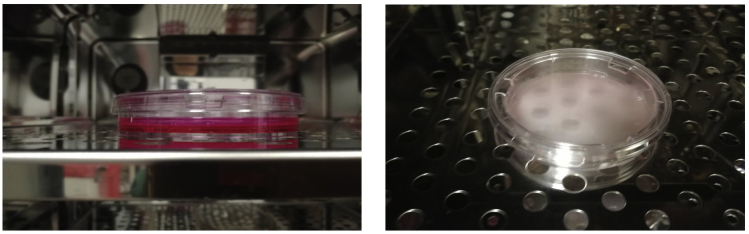
Cell attachment to Transwell filters Cells seed on top of Transwell filters are allowed to attach to the filter for about 16 h.

Methods video S1. Careful seeding of cells on Transwell filter top, related to step 3

### Protrusion induction and purification - day 2

**Timing: 3–4 h**

Protrusions are induced in a synchronized manner by adding media to the bottom chamber. Protrusions and cell bodies are then fixed and collected in lysis buffer.

***Note:*** See Methods video S2 for steps 4–6.

4. Carefully remove Transwell from the incubator and remove media by aspiration.
5. Add 8 mL of fresh media to the top of filter and 12 mL fresh media to the bottom chamber. This will open the pores to the cells and induce the formation of protrusions.

***Note:*** Media can be added to the bottom chamber through the three openings on the sides of filter inserts.

6. Allow protrusion formation by placing Transwell in the incubator for the desired length of time.

***Note:*** We perform a physical protrusion formation stimulation. For other methods based on chemotactic stimulation please see (Mili et al., 2008).

***Optional:*** Different incubation times may be used, which may give a different protrusion proteome profiles as we showed in Dermit et al. (2020). In the experiment shown here, we performed a time course of 30, 60, 120, 240, and 480 min.

***Note:*** See Methods video S3 for steps 7 and 8.

Methods video S2. Replace fresh media on the top of the filter and add media to the bottom, related to steps 4–6

7. Remove media and wash off the remaining media with PBS.
8. Remove PBS and add ice-cold methanol (8 mL to the top and 12 mL to the) to the filter. Place Transwells with methanol for at least 20 min at −20°C.

***Note:*** Whilst methanol fixation is not necessary if just one filter is being processed, fixation allows processing of multiple Transwells together at the same time. After fixing, the filter can stay in methanol at −20°C for as long as necessary, allowing one-by-one collection of protrusion and cell-bodies.

***Note:*** See Methods video S4 for steps 9–11.

Methods video S3. Remove media, wash with PBS, and add cold methanol, related to steps 7 and 8

9. Place the dish on ice, remove the methanol and gently re-hydrate by adding ice-cold PBS. Aspirate the PBS thoroughly.

***Note:*** The membrane shape will change due to the dehydration/rehydration.

10. Place 100 μL of lysis buffer inside the lid of a 50 mL falcon tube. Dip a glass coverslip in the lysis buffer and gently shave protrusions off the bottom surface of the filter using the dipped cover slip. Repeat until the whole surface area is shaved.
11. Once protrusions are collected, collect the cell-body fraction by directly adding 500 μL of lysis buffer to the top of the filter. Pipette the lysis buffer over the whole surface area multiple times to lyse as much cell-body as possible.

***Note:*** Protrusion and cell body fractions are collected in protein LoBind® tubes to prevent protein loss.

***Note:*** Incompletely solubilized or too viscous lysates can be sonicated. We routinely sonicate MDA-MB231 lysates on Bioruptor Pico sonication device and perform 10 cycles of 30 sec ON and 30 sec OFF. Cell bodies tend to be more viscous due to presence of DNA.

**Pause point:** The lysates can be stored at −20°C for several weeks, or at −80°C for several months. A pilot test with Sodium Dodecyl Sulphate Polyacrylamide Gel Electrophoresis (SDS-PAGE) and Coomassie blue staining could be run to estimate protein integrity.

Methods video S4. Remove methanol, wash with PBS, and collect protrusions, related to steps 9–11

### Sample preparation for proteomics - day 3

**Timing: 5–6 h**

Protein amounts are estimated and samples are prepared for protein digestion.

***Note:*** All steps are performed at 20°C–23°C unless indicated otherwise.

12. Estimate protein amounts by BCA assay according to manufacturer's instructions. Use multiple replicate reactions per sample and standards to improve the accuracy of measurements.
13. Transfer 25 μg of each sample to a fresh tube.
14. Add Bond-Breaker TCEP Solution reducing agent to a final concentration of 50 mM (1/10 dilution), and incubate at 95°C for 10 min. Spin down for 30 sec at 16,000 *g* to collect any condensation.
15. Allow the samples to cool down to RT. Add 7x volumes of UA buffer to each reduced sample, and mix gently by inverting the tube multiple times. For example, if sample volume is 50 μL (for 25 μg of sample); add 389 μL of UA buffer [ (50 μL of sample + 5.5 μL of TCEP Solution) ∗7].
16. Transfer to Vivacon 500 Hydrosart filters with a molecular cut-off of 30 kDa, for buffer exchange (McDowell et al., 2013). Concentrate samples by centrifugation at 14,000 *g* for 20 min. Discard the flow-through.

***Note:*** A 30 kDa cut-off filter efficiently removes SDS micelles that have an average size of ∼20 kDa. On the other hand, since denatured proteins assume a much larger volume than their folded state, a 30 kDa cut-off filter can still effectively retain most denatured proteins that are >10 kDa in size (Wisniewski, 2019).

**CRITICAL:** Seldom times Vivacon 500 Hydrosart filters happen to have a faulty seal. To avoid losing material, perform a 1 min quick centrifugation and check how much solution has gone through to the collection tube. Normally, this should be only a small fraction after a short spin, but all material will go through if the seal is faulty. In such case, transfer the sample to a new Vivacon 500 Hydrosart filter.

***Note:*** Maximum capacity of Vivacon 500 Hydrosart filters is 500 μL. If volume surpasses the capacity repeat the loading step and centrifugation until all volume has been loaded onto the filter.

***Note:*** Sometimes it can take longer for the solution to pass through the filter, especially if too viscous. If the solution has not completely gone through the filter in 20 min, centrifuge for longer until almost all of the solution has gone through. A small amount (∼5–10 μL) remaining on the filter is fine.

17. Wash the filters twice with UA buffer through cycles of 400 μL buffer addition and concentration at 14,000 *g* for 20 min. Discard the flow-through.
18. Alkylate samples by adding 10 mM iodoacetamide in UA buffer to the filters and incubating at RT for 30 min in the dark.

***Note:*** Iodoacetamide is light sensitive. Make fresh and keep the solution protected from light at all times.

19. Concentrate by centrifuging at 14,000 *g* for 10 min.
20. Add 200 μL of UA buffer to the filters and concentrate again by centrifuging at 14,000 *g* for 20 min. Discard the combined flow-through. Repeat twice.
21. Add 100 μL of 100 mM Triethylammonium bicarbonate (TEAB) to each filter and concentrate by centrifuging at 14,000 *g* for 10 min. Repeat twice. Discard the combined flow-through.

***Note:*** TMT reagents are amine-reactive; non-amine-based buffer such as TEAB or HEPES are therefore required for efficient labelling (see step 25).

21. Transfer the filters to new collection tubes. Add 100 μL of 100 mM TEAB buffer to each filter.
22. Immediately before use, thaw a sequencing-grade trypsin vial and add 0.5 μg trypsin to each filter. Incubate filter for about 16 h at 37°C in the thermomixer with shaking (600 rpm).

***Note:*** To stop filters from drying out, double seal the lid and spin columns with parafilm.

***Note:*** A trypsin/ substrate ratio between 1:100 to 1:20 (w/w) is recommended.

***Note:*** Trypsin digestion efficiency for each experiment can be checked *a posteriory*. See troubleshooting 3.

### Isobaric peptide labeling - day 4

**Timing: 2.5 h**

**Tryptic** **peptides are labeled with TMT isobaric mass tags**

23. Bring TMT10plex^TM^ isobaric label reagent kit stored at −20°C to 20°C–23°C.

***Note:*** TMT10plex^TM^ kit contains 0.8 mg per labelling channel. 0.2 mg of each channel is sufficient to label 25 μg of protein. Each label vial can be used for 4 independent experiments.

24. Add 164 μL of acetonitrile to each label vial for reconstitution, and incubate for 5 min with occasional vortexing to dissolve.
25. Briefly centrifuge the label vials to collect the solutions. Label each filter for about 16 h digested sample with a specific TMT label by adding 41 μL of a given label solution to a Vivacon filter.

***Note:*** Make sure to record which TMT label you add to which sample. In this experiment we labeled protrusions after 30 min induction with TMT10-127N tag and corresponding cell bodies with TMT10-126 tag; protrusions after 60 min induction with TMT10-128N tag and corresponding cell bodies with TMT10-127C tag; protrusions after 120 min induction with TMT10-129N tag and corresponding cell bodies with TMT10-128C tag; protrusions after 240 min induction with TMT10-130N tag and corresponding cell bodies with TMT10-129C tag; protrusions after 480 min induction with TMT10-130C tag and corresponding cell bodies with TMT10-131N tag. Incubate the reaction for 1 h at 25°C in thermomixer with shaking (600 rpm) to label the peptides.

***Note:*** TMT label efficiency can be checked *a posteriory*. See troubleshooting 4.

26. Add 50 μL of the Quenching Reagent (50% hydroxylamine) to 450 μL of 100 mM TEAB and mix by vortexing. Then quench the TMT-labeling reaction by adding 8 μL of this diluted quenching solution to each Vivacon filter.
27. Incubate for 30 min at 25°C in the thermomixer with shaking (600 rpm).
28. Elute the TMT-labeled peptides by centrifuging the filters at 14,000 *g* for 10 min.
29. Without removing the eluate from the collection tubes, perform an additional elution by adding 40 μL of 100 mM TEAB and centrifuging the filters at 14,000 *g* for 10 min. Repeat once. Keep the combined eluate in the collection tubes.
30. Without removing the eluate from the collection tubes, perform a final elution by adding 40 μL of 30% Acetonitrile and centrifuging the filters at 14,000 *g* for 10 min.
31. Combine the differentially TMT-labeled eluates from individual filters in one tube and lyophilize in a speedvac.

***Note:*** We operate our speedvac at ambient temperature. The running time for drying combined TMT 10plex samples is >5 h. Samples can be left drying in speedvac for about 16 h.

### High pH fractionation - day 5

**Timing: 1.5 h**

To increase the proteomic detection depth, TMT-labeled peptide mixes are fractionated using High pH reversed-phase peptide fractionation spin columns.

32. Resuspend 100 μg of TMT-labeled peptides in 300 μL of 0.1% Trifluoroacetic acid (TFA). Load the peptides into a pre-conditioned spin column from the High pH reversed-phase peptide fractionation kit by centrifugation (3000 *g*, 2 min). See manufacturer’s instructions for column conditioning.
33. Wash the column once with LC-MS grade H_2_O by centrifugation (3000 *g*, 2 min).
34. Prepare a step gradient of increasing acetonitrile concentrations (5%, 10%, 12.5%, 15%, 17.5%, 20%, and 25%) in 0.1% triethylamine, included in the fractionation kit. Progressively apply each of these mixtures to the column and elute by centrifugation (3000 *g*, 2 min). Collect eluates and keep separately (i.e., 7 different fractions).
35. Lyophilize each fraction eluate in a speedvac.

***Note:*** Samples may be left drying in the speedvac for about 16 h.

**Pause point:** The lyophilized samples can be stored at −20°C or −80 for several weeks.

### Mass spectrometry injection, analysis, and data collection - day 6

**Timing: 2 days**

Dried peptides are reconstituted and injected in the mass spectrometer.

36. Reconstitute samples in 0.1% formic acid (FA).

***Note:*** Reconstitution volume may depend on the amount and number of samples, as well as the mass spectrometry instrument used. For example, for 100 μg of mixed peptides, we would expect to have ∼14 μg (100/7) in each fraction on average. Therefore, we would reconstitute in 10 μL of FA and inject 1 μL ( ∼1.4 μg) into the nanoflow HPLC for analysis on the Q-Exactive™ Plus mass spectrometer.

37. Centrifuge the samples at 4°C at 12,000 rpm for 10 min to remove any insoluble debris, and transfer the supernatant to a 0.2 low binding tube inside an autosampler vial. Place the vial in the LC autosampler and inject the required amount for LC-MS analysis.
38. Parameters used for LC-MS/MS system are listed in Table 2.

**Table 2:** .Parameters for LC-MS/MS

Action	Characteristics
Resolution	Flow rate of 250 nl/min on an Easy-Spray 50 cm X 75 μm RSLC C18 column
Gradient	123 min gradient of 3% to 35% of Buffer B (0.1% FA in Acetonitrile) against Buffer A (0.1% FA in LC-MS gradient water)
Infusion	Electrospray ionization
Spray voltage	1.95 kV
Capillary Temp	255°C
Acquisition mode	Data dependent positive mode, top 15 method
Survey spectra	Full scan survey spectra (m/z 375–1,500)
Resolution	70,000 resolution for MS scans and 35,000 for the MS2 scans
Dynamic exclusion	30 s for fragmented peaks
39. Multiple injections can often be performed as technical replicates. We ran two technical replicates that generated 14 raw files [data deposited in PXD015808 and shown in Dermit et al. (2020)].

## Quantification and statistical analysis

**Timing: 1 day**

Analysis of mass spectrometry proteomics data.

**Figure 4:**
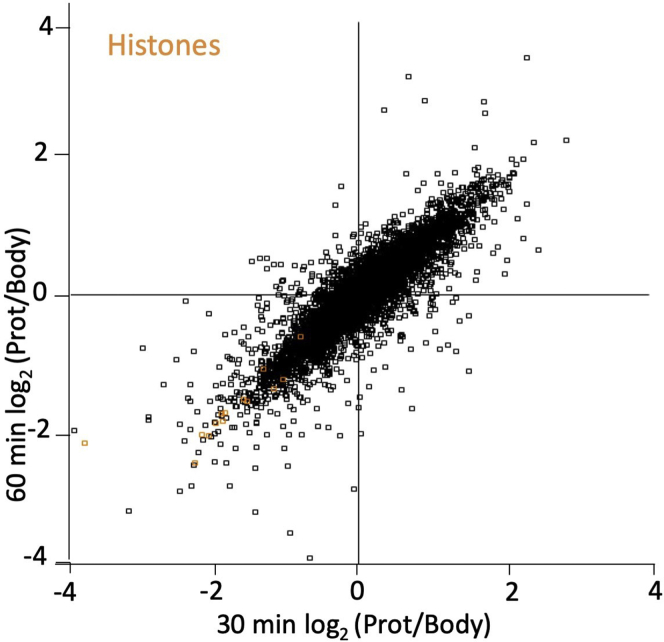
Scatter plot of log_2_(protrusion/cell bodies) ratio values from samples collected after 30 min and 60 min of protrusion induction, plotted against each other Highlighted in orange are histone proteins, enriched in the cell body fraction.
  i. On the “Processing” section, select “Annotation column” and “1D annotation enrichment”. Drag column samples to analyze test to the whether the corresponding expression values to every categorical term has a preference to be systematically larger or smaller than the global distribution of expression values (Figure 6J) (Table S2).
    ***Note:*** In this experiment the category with highest score is “cytoplasmic translation” for 240 min timepoint.
    ***Note:*** Perseus files can be saved as .sps file. The Perseus file generated in this manuscript can be found here https://github.com/demar01/STAR-Protocols.

***Note:*** You may want to assess data quality prior to analysis using tools like RawMeat, a tool designed for Thermo instruments to check data quality (http://proteomicsresource.washington.edu/protocols06/RawMeat_1007.exe) or other software tools (Bielow et al., 2016). However, visualisation of the searched and processed data in form of scatter plots (Figure 4), provides a better way to assess the data quality and the overall success of the experiment.

**Figure 5:**
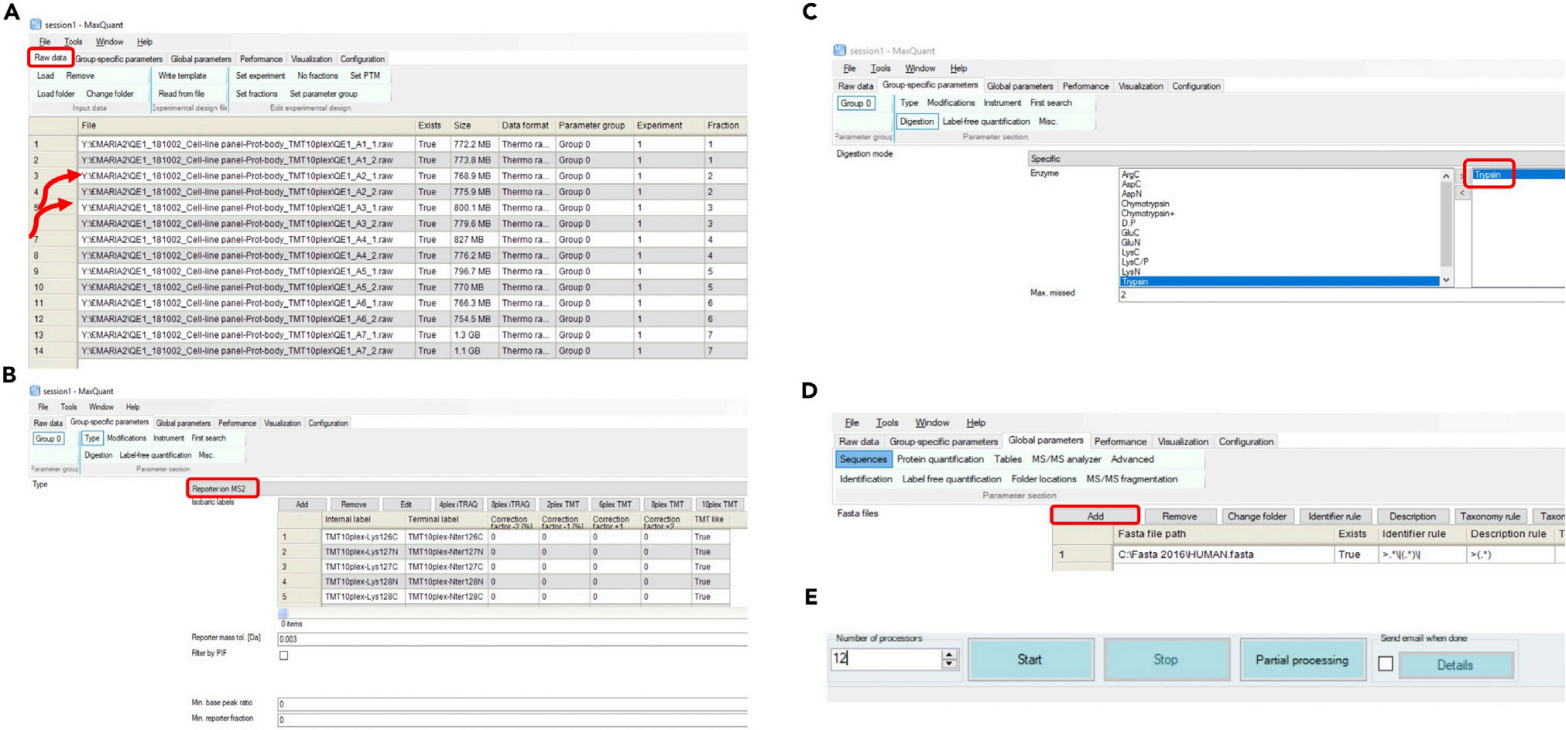
Screenshots of MaxQuant workflow followed to perform analysis of LC-MS/MS raw data of cell bodies and protrusions (A) Load raw mass spectrometry data contained in a given folder. (B) Select ‘Reporter ion MS2’ and choose the preconfigured TMT10plex isobaric labels. (C) Select trypsin as protease and allow for up to two missed cleavages. (D) Add human FASTA file to match this theoretical spectrum against the measured spectra. Select Uniprot identifier. (E) Select the number of threads. One thread corresponds to one unit of processor and additionally needs 2 GB of RAM for processing, so if your computer has 4 CPU cores and 8 GB RAM, you can increase the number of processors to a maximum of 4.

**Figure 6:**
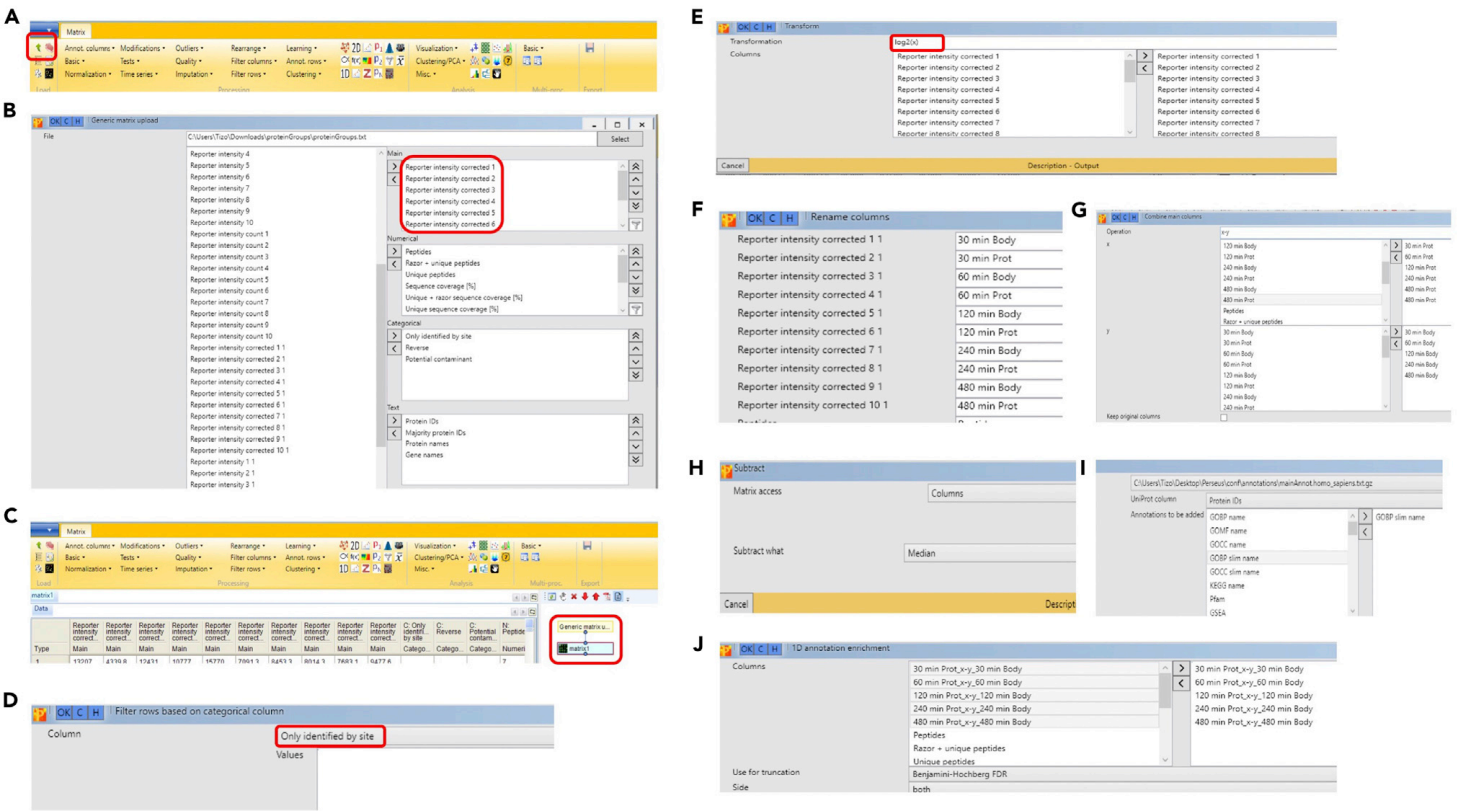
Screenshots of Perseus workflow followed to perform data analysis of MaxQuant proteinGroups.txt output (A) Tab-separated files can be loaded into Perseus by clicking into small green arrow button on the upper left corner. (B) Perseus window for loading proteinGroups.txt file with identification and quantification data. (C) Loaded data appear in Perseus as a matrix containing all protein identifications in rows and with experiments as columns. (D) Selecting “Filter rows by categorical column” opens a new window for selecting the types of protein entries (proteins identified by site, contaminants, and reverse hits) to be eliminated from the dataset. (E) Selecting “Transform”, apply log_2_ transformation of intensity values. (F) To rename columns manually, select “Rename Column” and type the new names in the predefined text field. (G) Selecting “Combine main columns” pairs of body and protrusion columns are combined into single columns by subtracting protrusion Log values from those of cell-body. Please make sure that the order numbers of columns that are selected in the 'x' and 'y' box are equal. (H) Selecting “Normalization” and “Subtract” to normalize the intensity values by column. For each protein intensity subtract the median expression within each sample. (I) Selecting adding annotation, use “mainAnnot.homo_sapiens.txt.gz” as source. Select which annotation is added to the current matrix (here we select “GOCC slim name”) transferring it to the right-hand side. (J) Selecting “Annotation columns” and “1D annotation enrichment” to test whether the corresponding expression values to every categorical term has a preference to be systematically larger or smaller than the global distribution of expression values.

1. Use MaxQuant computational platform for analysis of mass spectrometry data.
  ***Note:*** Please see Figure 5 for workflow followed on MaxQuant computational platform for analysis of these data. Unless explicitly stated, parameters in MaxQuant have not been changed from their default values.
  ***Note:*** For complete details on the use and execution of MaxQuant platform for mass spectrometry-based proteomics search and quantifications, please refer to (Tyanova et al., 2016).
  a. On the “Raw data” tab select “Load folder” to load all generated files on this experiment onto MaxQuant. We are working with 14 raw files that are ∼ 700 MB- 1.3 GB each (Figure 5A).
  b. Assign experiment number and fractions (Figure 5A).
  c. On the “Group-specific parameters” tab select reporter ion MS2 and TMT10plex. (Figure 5B).
  d. On the same tab select Trypsin enzyme, allowing a maximum of 2 missed cleavages (Figure 5C).
  e. On the “Global parameter” tab add a FASTA file of the Homo sapiens proteome. We extracted human proteome from Uniprot (The UniProt, 2017) (Figure 5D).
    ***Note:*** In ‘razor’ peptide assignment, the peptide is assigned to the protein group with the larger number of identified peptides or, in case of a tie, to the protein group with more highly scoring peptides.
  f. Select the number of threads or processors. Each thread needs at least 2 GB of RAM. The number of threads should be equal to or fewer than the number of logical cores available on your computer, or MaxQuant may crash. Our computer has 12 cores with >24 GB of RAM so we select 12 threads. Click the “Start” button (Figure 5E).
    ***Note:*** The time that it will take MaxQuant to process the raw files varies greatly depending on the computing power available. The speed of the hard-drive is also an important factor. For the experiment discussed here, the job took ∼6 h on our PC when 12 cores were utilized.
    ***Note:*** proteinGroups.txt file output of MaxQuant can be found in Table S1.

2. Use Perseus platform for integrative analysis of proteomics data.
  ***Note:*** Please see Figure 6 for workflow followed on Perseus platform for further analysis of these data.
  ***Note:*** For complete details on the use and execution of Perseus, please refer to (Tyanova and Cox, 2018).
  a. Once search is finished, on the “Processing” section of Perseus, click “Generic matrix upload” (on the top left-hand size, represented by a green arrow, Figure 6A). Select the MaxQuant output proteinGroups.txt file. Transfer “Reporter intensity corrected” expression columns into the “Main” column window on the right—hand side, and the columns containing identifiers (e.g., protein IDs) into the “Text” column window. Press the OK button (Figure 6B).
    ***Note:*** The data will be loaded into Perseus workflow panel as matrix 1 (Figure 6C).
  b. On the “Processing” section select “Filter rows” and select “Filter rows based on categorical column” to exclude proteins identified by site. Repeat this step to exclude proteins matching to the reverse database or contaminants (Figure 6D).
  c. On the “Processing” section select “Basic”, click “Transform” and specify the transformation function log_2_(x). Log transforming the intensity values is crucial for unbiased processing when intensities are to be converted into ratio values (Figure 6E).
  d. On the “Processing” section select “Rearrange” and click on “Rename columns” to specify new names for expression columns. We rename columns to “30 min Body”, “60 min Body”, “120 min Body”, “240 min Body”, “480 min Body”, “30 min Prot”, “60 min Prot”, “120 min Prot”, “240 min Prot” and “480 min Prot” (Figure 6F).
  e. On “Processing” section, select the “Basic” menu and click on the “combine main columns” button. Set operation to x-y (default) and transfer protrusions samples to x section on the top right corner and body samples to y section on the bottom right corner. This function will result in log_2_(protrusion/cell body) ratio values for each condition (Figure 6G).
    **CRITICAL:** You must ensure that protrusion and bodies across replicates are transferred in the correct order into the comparison panel. See Figure 6G.
  f. On “Processing” section, select “Normalization” to median normalize the ratio values. Select matrix access “Column” and subtract “median”. This will subtract the median of each column from each value (Figure 6H). Median normalization eliminates experimental variations due to unequal sample loadings by assuming that the proteome as a whole has no localization bias and its median is therefore distributed 1 to 1 between protrusions and cell-bodies.
  g. On the “Processing” section, select “Annotation column” to add protein specific annotations [such as GOBP (Ashburner et al., 2000)] (Figure 6I).
  h. On the “Analysis” section, select “Visualization” and select “scatter plot” to plot technical replicates against each other. Explore protein distribution across samples and distribution of known markers. For example, nuclear proteins such as histones should be enriched in the cell body fractions, whilst actin binding proteins should show enrichment in protrusion (Figure 4).
    ***Note:*** If working with 3 or more replicates/cell lines, one could also perform one sample t test to see which protein groups are significantly changed between protrusion and cell bodies. This test checks which protein groups are significantly different from a fixed value [e.g., 0 for log_2_ (Prot/body), meaning 1 to 1 distribution] upon setting a given threshold for p-value (e.g., 0.05). This would add 3 additional columns to the matrix: one containing the t test p value (-Log t test p value), other containing the t test difference, and finally a categorical column where the symbol '+' appears when the variation between the groups is statistically significant with respect to the specified threshold. This output could be used to generate a volcano plot where x axis contains the t test difference and y axis contains the–log t test p value. A similar approach was presented by Dermit et al. (2020).

## Expected outcomes

With our LC-MS/MS and MaxQuant settings we quantified a total of 5541 proteins (see troubleshooting 2, 3, and 4 if the number of identified/quantified proteins is low). Proteins such as actin binding proteins are expected to be enriched in protrusions, whilst nuclear proteins such as histones are expected to be depleted. Perform a quality control analysis by plotting the distribution of these expected protein categories (Figure 4). See troubleshooting 5 for potential solutions if expected distribution of proteins does not match observed protein distribution.

## Limitations

**Figure 9:**
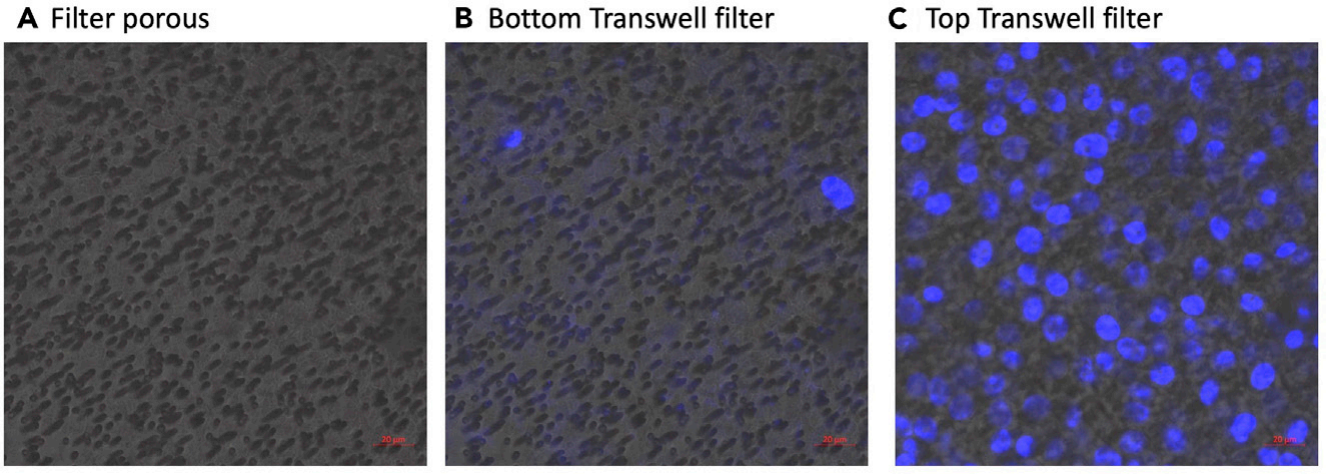
Representative confocal images of collagen-I-coated 3-μm pores (A, arrows) we observe clusters of pores on the porous filter (B) some nucleus (stained with HCS NuclearMask, H10325) can appear in the filter bottom, especially if protrusions are left to form for a long time. In this representative image pores were left open for 8 h. (C) most cells remain in the top of the filter chamber. Z-planes between lower and upper sides are ~ 10 μm.

This protocol describes protrusion profiling using 75 mm Transwells with 3 μm pores. These Transwells are often back ordered so expect long delivery times when ordering a new package. We have also noticed batch to batch variations in the distribution of filter pores which can affect the efficiency and specificity of protrusion purifications (See Figure 9, troubleshooting). Moreover, the pore size may have a great impact on profiled protrusions as suggested by (Wang et al., 2017). Therefore, the results obtained following this protocol may be specific to these settings. This protocol was specifically chosen as it mimics protrusions formation during cell migration in 3D pepsinized collagen-I matrix settings (Mardakheh et al., 2015).

## Troubleshooting

### Problem 1

Media goes through the filter after 16 h (when loading cells on top of microporous filter, step 3)

### Potential solution

Transwells are permeable supports when there is an equilibrium between the filter upper and lower compartments. If no media is added to the bottom compartment this equilibrium is not established yet. If too much media is added on top of Transwell, gravity will push media through pores.

Make sure that cells are seeded in a maximum 3.5 mL of media, the Transwell insert is properly fit into the plate chamber, and the incubator shelf is level.

### Problem 2

Little protein identification/quantification due to sample loss

### Potential solution

Protein loss during filter aid sample preparation can be a cause of damaged Vivacon filters. Make sure to perform a first spin check (on step 16). Vivacon filters can also clogged due to high viscosity of samples. Pre-sonication of samples (step 11) should improve recovery of all proteins. In addition, make sure that the correct mass-spectrometer method is used, optimize LC gradient to maximize MS/MS of unique peptides and ultimately and optimize sample ion injection.

### Problem 3

Little protein identification/quantification due to inefficient trypsin digestion.

### Potential solution

**Figure 7:**
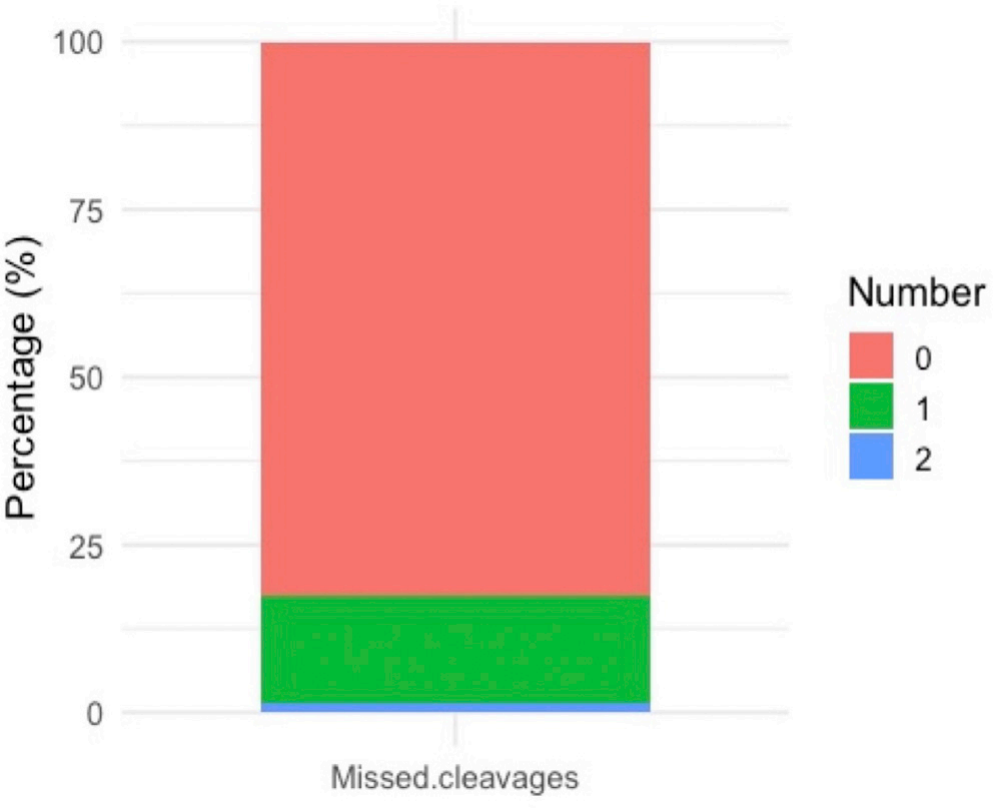
Percentage of missed cleavage peptides Protease efficiency can be calculated by the number of missed cleavages. Note that a maximum number of missed cleavages are normally set on the MaxQuant searches (for this experiment we allowed a maximum of two missed cleavages). Most of the tryptic peptides of this experiment have no missed cleavage.

After MaxQuant analysis, the number of enzyme missed cleavage sites is recorded in “peptides.txt”. Visualization of missed cleavages with *protrusionproteome::plot_miscleavagerate* function reveals the enzyme efficiency (step 22). Figure 7 shows that trypsin efficiency of this experiment was high since there are low number of missed cleavage sites. Low trypsin efficiency would be if 50% of peptides have more than one missed cleavage sites.

Inefficient trypsin digestion could be due to addition of insufficient enzyme, old/poorly stored enzyme, or incorrect digestion conditions. Acquire a fresh batch of trypsin and make sure it is reconstituted and stored according to manufacturer’s recommendation. Add the correct amount (1:20–1:100 w/w) and ensure that the digestion buffer pH is between 8–8.5. Using additional proteases with complementary cleavage specificity may also improve protein identifications (Dau et al., 2020).

### Problem 4

Little protein identification/quantification due to inefficient TMT labeling.

### Potential solution

**Figure 8:**
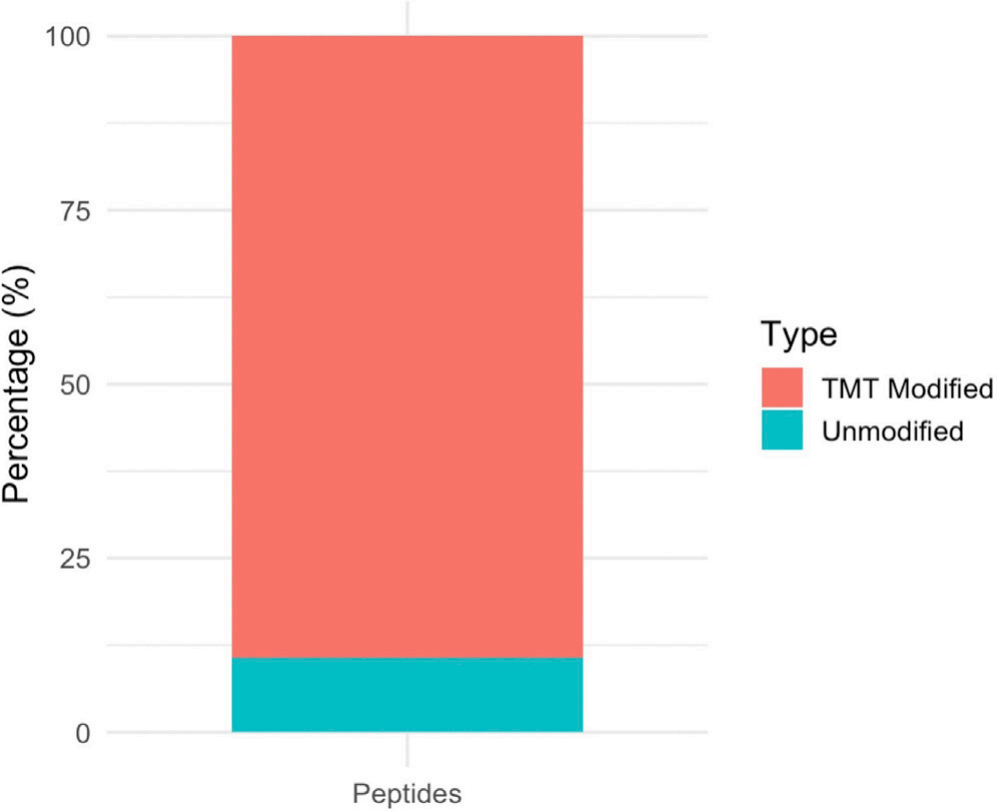
Percentage of TMT labeling efficiency This efficiency is obtained from the number of modifications recorded in the “evidence.txt” output of a separate MaxQuant search where TMT was set as variable modifications and not isobaric labels (Erdjument-Bromage et al., 2018).

To check TMT labeling efficiency, a separate search needs to be performed, using TMT as variable modifications rather than isobaric labels (Erdjument-Bromage et al., 2018). After this new MaxQuant search, the number of post-translational modifications within the peptide sequences is recorded in “evidence.txt”. Visualization of TMT incorporation with *protrusionproteome::plot_labelingefficiency* function reveals that the TMT labeling incorporation efficiency of all the N-terminal and lysine amine groups in peptides of this experiment was high, meaning that at least ∼90% labeling efficiency was achieved (to determine TMT labeling efficiency, step 25) (Figure 8).

There are several causes that may lead to poor TMT peptide labeling, therefore make sure that: a) do not exceed the amount of sample/TMT reagent; a minimum of 0.2 mg of each TMT label reagent is required to label 25 μg of protein, b) make sure that a non-amine-based buffer (e.g., TEAB) is used and pH is between ∼8–8.5, c) use neat acetonitrile or ethanol to reconstitute TMT tags as moisture can inactivate TMT labels, d) store lyophilized TMT reagents at −20°C and store reconstituted reagents at −80°C and use them within one week of reconstituting.

### Problem 5

Nuclear proteins are not depleted in the protrusion fraction (when checking expected protein distribution, step 2 h and in *protrussionproteome::plot_scatter function*)

### Potential solution

There are several reasons why nuclear proteins may be found in the protrusion fraction. Most likely of them is that rather than just forming protrusions, cells may have fully migrated through the filter pores. To rule out that cells can fully go through the pores, you can perform a fluorescence microscopy experiment on your filters after protrusion induction and stain the nucleus with DAPI. Nucleus should only be present on top of the filters (Figure 9). If you observe nuclei on the bottom side, this indicates that cells have migrated through. Several reasons may cause this. These include cell-type variations (some cells can squeeze their nuclei through very small pore sizes whilst others cannot), the length of time the cells are allowed to form protrusions, batch problems with filters, or incomplete drying of filters after collagen coating. If this problem persists, you may want to consider switching to Transwells with a smaller pore size and/or change the protrusion collecting time.

**Figure 10:**
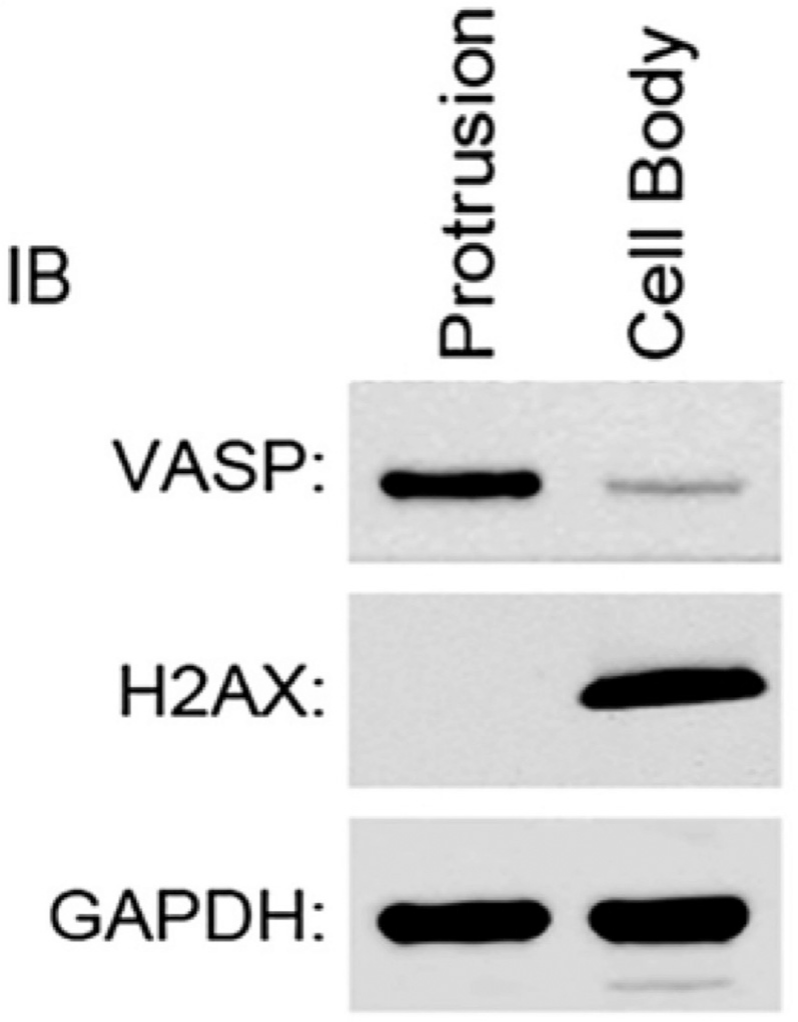
Validation of protrusion purification method by western blotting VASP, protrusions marker; H2AX, cell bodies marker; GAPDH, loading control. Figure was taken from Mardakheh et al. (2015) with permission.

In addition, if collection is not done properly (e.g., if cell bodies are collected before protrusions), you may be contaminating your protrusion fraction with solubilized proteins from the cell bodies. The quality of the fractionation can be assessed by western blot in a pilot experiment (Figure 10, troubleshooting).

## Resource availability

### Lead contact

Further information and requests for resources and reagents should be directed to and will be fulfilled by the lead contact, Faraz K Mardakheh (f.mardakheh@qmul.ac.uk).

### Materials availability

Cell line used in this study could be made available upon request to lead contact.

### Data and code availability

The mass spectrometry raw files and their associated MaxQuant output files generated during this study are available at ProteomeXchange Consortium (Vizcaino et al., 2014) via the PRIDE partner repository (http://www.ebi.ac.uk/pride/archive/), as listed in the Key Resources Table.

*All the functions to perform this analysis programmatically can be found in Protrusionproteome, an R package* (Wickham, 2009) available for download at https://github.com/demar01/protrusionproteome. Website for this package can be found here https://demar01.github.io/protrusionproteome/. Perseus .sps file can be found in https://github.com/demar01/STAR-Protocols.

## Supplemental information

Table S1. proteinGroups file output of MaxQuant produced in this study, related to quantification and statistical analysis step 1f

Table S2. Matrix containing the list of terms from categorical values included in GOBP annotations that are significantly biased towards high or low values in the expression columns, related to quantification and statistical analysis step 2i
